# Preoperative Risk Stratification in Arthroscopic Rotator Cuff Repair: Aspartate Aminotransferase-to-Platelet Ratio Index as an Estimate of Liver Dysfunction

**DOI:** 10.7759/cureus.41980

**Published:** 2023-07-16

**Authors:** Steven H Liu, Vaidehi Patel, Rachel A Loyst, Brandon Lung, Dorian Cohen, Kevin Kashanchi, David E Komatsu, Edward D Wang

**Affiliations:** 1 Department of Orthopaedic Surgery, Stony Brook University, Stony Brook, USA; 2 Department of Orthopaedic Surgery, University of California, Irvine, Orange, USA; 3 Department of Orthopaedic Surgery, University of Southern California, Los Angeles, USA

**Keywords:** complications, apri, fibrosis, cirrhosis, liver, shoulder, arthroscopy, arthroscopic rotator cuff repair

## Abstract

Background: Aspartate Aminotransferase-to-Platelet Ratio Index (APRI) is a cost-effective and noninvasive measure of liver function, an alternative to the gold standard liver biopsy which is resource-intensive and invasive. This study investigates the association between various degrees of liver dysfunction based on APRI and 30-day postoperative complications following arthroscopic rotator cuff repair (aRCR).

Methods: The American College of Surgeons National Surgical Quality Improvement Program database was queried for all patients who underwent aRCR between 2015 and 2021. The study population was divided into four groups based on preoperative APRI: normal/reference (APRI ≤ 0.5), mild fibrosis (0.5 < APRI ≤ 0.7), significant fibrosis (0.7 < APRI ≤ 1), and cirrhosis (APRI > 1). Multivariate logistic regression analysis was conducted to investigate the connection between preoperative APRI and postoperative complications.

Results: Compared to normal liver function, mild fibrosis was significantly associated with male gender, lower BMI, American Society of Anesthesiologists (ASA) classification ≥ 3, and comorbid diabetes, hypertension, chronic obstructive pulmonary disease, and bleeding disorders. Significant fibrosis was significantly associated with male gender, greater BMI, ASA classification ≥ 3, and comorbid diabetes, hypertension, and bleeding disorders. Cirrhosis was significantly associated with younger age, ASA classification ≥ 3, smokers, and comorbid diabetes and bleeding disorders. Compared to normal liver function, fibrosis was not associated with complications, significant fibrosis was associated with myocardial infarction, and cirrhosis was associated with major complications, sepsis, non-home discharge, and mortality. However, mild fibrosis, significant fibrosis, and cirrhosis were independently associated with any adverse 30-day postoperative complications following aRCR.

Conclusion: Among those with predicted liver damage based on preoperative APRI, 30-day postoperative complications following aRCR were not found to be independently associated with preoperative mild fibrosis, significant fibrosis, or cirrhosis. Our results suggest that APRI predictive of liver dysfunction may be a weaker deterrent to undergoing aRCR compared to other orthopedic surgeries.

## Introduction

Arthroscopic rotator cuff repair (aRCR) continues to be a successful method to treat rotator cuff injury and disease, a common cause of disability and pain in the United States [1-3]. From 1996 to 2006, there has been 600% increase in aRCRs in the United States, and studies have predicted that the incidence of aRCR will continue to rise [4,5]. Given this rapidly growing surgical volume of aRCR, identification of risk factors for complications following aRCR is becoming increasingly important to optimize outcomes and reduce patient morbidity associated with this procedure. The current literature identifies older age, female gender, smoking status, obesity, larger rotator cuff tear size, osteoporosis, tendon fragility, diabetes mellitus, and hypercholesterolemia as possible risk factors for postoperative complications following aRCR [6,7]. Preoperative laboratory abnormalities in serum albumin, hemoglobin A1c, thyroid stimulating hormone, and thyroxine have also been associated with complications following aRCR [8-10].

Liver dysfunction has previously been associated as a risk factor following various different orthopedic surgeries. For instance, patients with end-stage liver disease who undergo total joint arthroplasty (TJA) are three times more likely to experience any complication compared to patients with normal liver function [11]. In another study of patients who underwent tibia fracture fixation, patients with advanced cirrhosis were found to have a four-fold increased risk for 30-day readmission [12]. However, following aRCR, the relationship between liver dysfunction and complications remains unclear. Typically, liver function tests prior to non-hepatic surgery are recommended in patients with either diagnosed liver disease, clinical suspicion of liver dysfunction, or significant risk factors based on history and physical exam rather than all patients [13]. Using these preoperative laboratory studies, the aspartate aminotransferase (AST)-to-preoperative platelet ratio index (APRI) can be readily calculated to noninvasively estimate the degree of liver dysfunction. Preexisting literature has shown that APRI can distinguish between different levels of fibrosis and cirrhosis with good sensitivity and specificity [14,15].

This study aims to investigate the association between liver dysfunction as measured by preoperative APRI and 30-day postoperative outcomes in patients who underwent aRCR. We hypothesize that an abnormal preoperative APRI will predict higher rates of postoperative complications in patients who receive preoperative liver function studies. 

## Materials and methods

We queried the American College of Surgeons National Surgical Quality Improvement Program (ACS-NSQIP) database for all patients who underwent aRCR between 2015 and 2021. This study was exempt from approval by our University’s Institutional Review Board because the NSQIP database is fully de-identified. Data in the NSQIP database is obtained from over 600 hospitals in the United States and is collected by trained Surgical Clinical Reviewers. The data is periodically audited to maintain high fidelity. 

The Current Procedural Terminology (CPT) code 29827 was used to identify 47,601 patients who underwent aRCR between 2015 and 2021. After this, 34,630 patients with missing preoperative AST or platelet counts were excluded, leaving 12,971 patients. Cases were also excluded if any of the following variables had missing information: height/ weight (49 excluded), American Society of Anesthesiologists (ASA) classification (four excluded), and functional health status (116 excluded). A total of 169 patients were excluded, leaving a total of 12,802 patients to be included in this study. Using 40 as the upper limit of normal AST, we calculated the preoperative APRI for the remaining patients using the following formula [16]:

APRI = \frac{AST \times 100}{Upper\ limit\ of\ normal\ AST \times Platelet\ count\ (in\ thousands)}\\

**Figure 1:**
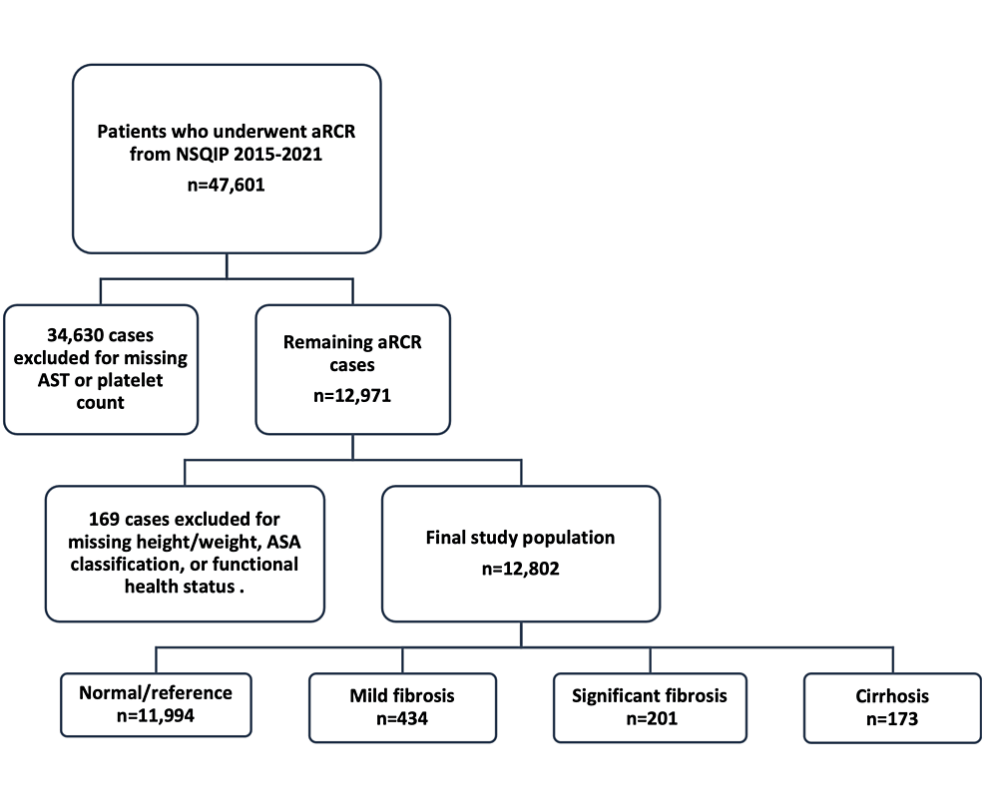
Case selection schematic. aRCR, Arthroscopic Rotator Cuff Repair; NSQIP, National Surgical Quality Improvement Program; ASA, American Society of Anesthesiologists; CPT, Current Procedural Terminology

The remaining study population (Figure 1) was then indexed into four cohorts based on their preoperative APRI: normal/reference (APRI ≤ 0.5), mild fibrosis (0.5 < APRI ≤ 0.7), significant fibrosis (0.7 < APRI ≤ 1), and cirrhosis (APRI > 1). APRI cutoff values of 0.5, 0.7, and 1 were chosen as validated threshold APRI given that previous studies have identified these cut-off values to be associated with a sensitivity and specificity of at least 74% and 49% for some liver damage, 77% and 72% for significant fibrosis, and 76% and 72% for cirrhosis, respectively [16].

Variables collected in this study included patient demographics, comorbidities, and 30-day postoperative complication data. Patient demographics included gender, BMI, age, smoking status, functional status, ASA classification, and preoperative steroid use. Steroid use status was defined as patients who routinely used immunosuppressants or corticosteroids within 30 days pre-procedure. Smoking status was defined as cigarette use at any point within the past year before the procedure. Preoperative comorbidities included congestive heart failure (CHF), diabetes, hypertension, severe chronic obstructive pulmonary disease (COPD), and bleeding disorders. Major and minor complications that occurred within 30 days postoperatively were included in the analysis. Major complications included the following: cardiac arrest requiring cardiopulmonary resuscitation, myocardial infarction, deep vein thrombosis requiring therapy, stroke, unplanned intubation, pulmonary embolism, failure to wean off a ventilator within 48 hours, sepsis, septic shock, deep incisional surgical space infection (SSI), and organ/space SSI, readmission, reoperation, mortality. Minor complications included the following: pneumonia, urinary tract infection (UTI), blood transfusions within 72 hours after surgery, wound dehiscence, and superficial incisional SSI.

All statistical analyses were conducted using SPSS Software version 26.0 (IBM Corp., Armonk, NY, USA). Patient demographics and comorbidities were compared between cohorts using bivariate logistic regression. Multivariate logistic regression, adjusted for all significantly associated patient demographics and comorbidities for the respective cohort, was used to identify associations between preoperative APRI and postoperative complications. Odds ratios (OR) were reported with 95% confidence intervals (CI). The level of statistical significance was set at p < 0.05. 

## Results

Compared to the normal APRI group, the mild fibrosis group was associated with male gender (p < 0.001), lower BMI groups (p = 0.002), ASA classification ≥ 3 (p < 0.001), and comorbid diabetes (p = 0.045), hypertension (p = 0.013), COPD (p = 0.031), and bleeding disorders (p < 0.001). Compared to the normal APRI group, the significant fibrosis group was associated with male gender (p < 0.001), greater BMI groups (p = 0.019), ASA classification ≥ 3 (p = 0.001), and comorbid diabetes (p = 0.001), hypertension (p < 0.001), and bleeding disorders (p < 0.001). Compared to the normal APRI group, the cirrhosis group was associated with younger age groups (p = 0.002), ASA classification ≥ 3 (p = 0.003), smokers (p = 0.028), and comorbid diabetes (p = 0.025) and bleeding disorders (p < 0.001) (Table 1).

**Table 1:** Patient demographics and comorbidities for patients with preoperative normal APRI, Mild fibrosis, significant fibrosis, and cirrhosis. Bold p values indicate statistical significance with p < 0.05. APRI, AST to Platelet Ratio Index; BMI, body mass index; ASA, American Society of Anesthesiologists; CHF, congestive heart failure; COPD, chronic obstructive pulmonary disease

	Normal (APRI ≤ 0.5)	Mild fibrosis (0.5 < APRI ≤ 0.7)		Significant fibrosis (0.7 < APRI ≤ 1)		Cirrhosis (APRI > 1)	
	Number (%)	Number (%)	p value	Number (%)	p value	Number (%)	p value
Overall	11,994 (100.0)	434 (100.0)		201 (100.0)		173 (100.0)	
Sex			<0.001		<0.001		0.716
Female	5,403 (45.0)	118 (27.2)		66 (32.8)		64 (37.0)	
Male	6,591 (55.0)	316 (72.8)		135 (67.2)		109 (63.0)	
Age			0.279		0.267		0.002
18-39	304 (2.5)	8 (1.8)		5 (2.5)		9 (5.2)	
40-64	7,350 (61.3)	278 (64.1)		131 (65.2)		119 (68.8)	
65-74	3,451 (28.8)	125 (28.8)		53 (26.4)		37 (21.4)	
≥75	889 (7.4)	23 (5.3)		12 (6.0)		8 (4.6)	
BMI (kg/m^2)			0.002		0.019		0.289
<18.5	42 (0.4)	139 (32.0)		1 (0.5)		49 (28.3)	
18.5-29.9	5,474 (45.6)	166 (38.2)		81 (40.3)		73 (42.2)	
30-34.9	3,412 (28.4)	72 (16.6)		46 (22.9)		31 (17.9)	
35-39.9	1,758 (14.7)	57 (13.1)		45 (22.4)		20 (11.6)	
≥40	1,308 (10.9)	0 (0.0)		28 (13.9)		0 (0.0)	
Functional Status Prior to Surgery			0.603		0.997		0.288
Dependent	61 (0.5)	3 (0.7)		0 (0.0)		2 (1.2)	
Independent	11,933 (99.5)	431 (99.3)		201 (100.0)		171 (98.8)	
ASA classification			<0.001		0.001		0.003
≤2	6,655 (55.5)	200 (46.1)		88 (43.8)		76 (43.9)	
≥3	5,339 (44.5)	234 (53.9)		113 (56.2)		97 (56.1)	
Smoker			0.348		0.890		0.028
No	10,282 (85.7)	379 (87.3)		173 (86.1)		138 (79.8)	
Yes	1,712 (14.3)	55 (12.7)		28 (13.9)		35 (20.2)	
Steroid use			0.514		0.563		0.559
No	11,542 (96.2)	415 (95.6)		195 (97.0)		165 (95.4)	
Yes	452 (3.8)	19 (4.4)		6 (3.0)		8 (4.6)	
Comorbidities							
CHF	62 (0.5)	0 (0.0)	0.997	2 (1.0)	0.361	2 (1.2)	0.261
Diabetes	2,609 (21.8)	112 (25.8)	0.045	63 (31.3)	0.001	50 (28.9)	0.025
Hypertension	6,572 (54.8)	264 (60.8)	0.013	138 (68.7)	<0.001	90 (50.2)	0.467
COPD	425 (3.5)	24 (5.5)	0.031	11 (5.5)	0.147	6 (3.5)	0.958
Bleeding Disorder	194 (1.6)	21 (4.8)	<0.001	15 (7.5)	<0.001	11 (6.4)	<0.001

Compared to the normal APRI group, the mild fibrosis group was not associated with any postoperative complications (Table 2). The significant fibrosis group was associated with myocardial infarction (p = 0.045). The cirrhosis group was associated with major complications (p = 0.044), sepsis (p = 0.042), non-home discharge (p = 0.008), and mortality (p = 0.011).

**Table 2:** Bivariate analysis of 30-day postoperative complications in patients with preoperative normal APRI, mild fibrosis, significant fibrosis, and cirrhosis. Bold p values indicate statistical significance with p < 0.05. APRI, aspartate aminotransferase-to-platelet ratio index; SSI, surgical space infection

	Normal (APRI ≤ 0.5)	Mild fibrosis (0.5 < APRI ≤ 0.7)		Significant fibrosis (0.7 < APRI ≤ 1)		Cirrhosis (APRI > 1)	
	Number (%)	Number (%)	p value	Number (%)	p value	Number (%)	p value
Major complications	181 (1.5)	7 (1.6)	0.112	5 (2.5)	0.267	6 (3.5)	0.044
Minor complications	76 (0.6)	1 (0.2)	0.362	1 (0.5)	0.810	1 (0.6)	0.927
Overall complications	194 (1.6)	8 (1.8)	0.294	5 (2.5)	0.581	7 (4.0)	0.054
Sepsis	8 (0.1)	0 (0.0)	0.560	0 (0.0)	0.999	1 (0.6)	0.042
Septic shock	1 (0.0)	0 (0.0)	1.000	0 (0.0)	1.000	0 (0.0)	1.000
Pneumonia	20 (0.2)	0 (0.0)	0.771	1 (0.5)	0.286	0 (0.0)	0.998
Reintubation	8 (0.1)	0 (0.0)	0.999	0 (0.0)	0.999	0 (0.0)	0.999
Urinary tract infection	33 (0.3)	1 (0.2)	0.884	0 (0.0)	0.998	1 (0.6)	0.464
Stroke	4 (0.0)	0 (0.0)	0.999	0 (0.0)	0.999	0 (0.0)	0.999
Cardiac arrest	3 (0.0)	0 (0.0)	0.999	0 (0.0)	0.999	0 (0.0)	0.999
Myocardial Infarction	7 (0.1)	0 (0.0)	0.482	1 (0.5)	0.045	0 (0.0)	0.999
Bleeding transfusions	3 (0.0)	0 (0.0)	0.999	0 (0.0)	0.999	0 (0.0)	0.999
Deep vein thrombosis	15 (0.1)	1 (0.2)	0.992	0 (0.0)	0.999	0 (0.0)	0.999
Pulmonary embolism	17 (0.1)	0 (0.0)	0.998	0 (0.0)	0.999	0 (0.0)	0.999
Failure to wean off ventilator	4 (0.0)	0 (0.0)	0.999	0 (0.0)	0.999	0 (0.0)	0.999
Deep incisional SSI	7 (0.1)	0 (0.0)	0.999	0 (0.0)	0.999	0 (0.0)	0.999
Superficial incisional SSI	17 (0.1)	0 (0.0)	0.998	0 (0.0)	0.999	0 (0.0)	0.999
Organ/space SSI	6 (0.1)	0 (0.0)	0.402	1 (0.5)	0.033	0 (0.0)	0.999
Wound dehiscence	4 (0.0)	0 (0.0)	0.999	0 (0.0)	0.999	0 (0.0)	0.999
Readmission	149 (1.2)	6 (1.4)	0.075	5 (2.5)	0.125	5 (2.9)	0.062
Reoperation	47 (0.4)	0 (0.0)	0.253	1 (0.5)	0.813	0 (0.0)	0.998
Non-home discharge	71 (0.6)	1 (0.2)	0.335	2 (1.0)	0.468	4 (2.3)	0.008
Mortality	4 (0.0)	0 (0.0)	0.241	0 (0.0)	0.999	1 (0.6)	0.011

After controlling for all significantly associated patient demographic and comorbidity factors, the mild fibrosis, significant fibrosis, and cirrhosis groups were not independently associated with any adverse 30-day postoperative complications following aRCR compared to normal liver function (Table 3).

**Table 3:** Multivariate analysis of 30-day postoperative complications in patients with preoperative normal APRI, mild fibrosis, significant fibrosis, and cirrhosis. Bold p-values indicate statistical significance with p < 0.05. Dashes represent variables not significant in bivariate analysis. OR odds ratio; CI, confidence interval

	Significant fibrosis (0.7 < APRI ≤ 1)	Cirrhosis (APRI > 1)
	OR, 95% CI; p-value	OR, 95% CI; p-value
Major complications	--	0.95, 0.80-1.13; 0.564
Sepsis	--	0.624, 0.25-1.54; 0.305
Myocardial infarction	4.251, 0.55-33.07; 0.167	--
Non-home discharge	--	0.825, 0.62-1.09; 0.177
Mortality	--	1.973, 0.62-6.31; 0.251

## Discussion

The present study investigated the association between preoperative liver function and 30-day postoperative outcomes following aRCR. We used APRI as a validated, non-invasive estimate of liver function [14-16]. We found that, in patients who received preoperative liver function testing, preoperative APRI values suggestive of mild fibrosis, significant fibrosis, and cirrhosis were not independently associated with a greater risk of any short-term postoperative complications.

Analysis of 12,802 patients who underwent aRCR between 2015 and 2021 revealed that patients with elevated preoperative APRI had higher rates of ASA classification ≥ 3, comorbid diabetes, and bleeding disorders. These findings concur with existing literature noting similar associations between these three patient characteristics and elevated APRI in other patient populations, such as patients undergoing non-hepatic surgery and patients with diabetes and hepatitis C [17-19]. Interestingly, while the current literature also notes a strong correlation between male gender and liver dysfunction, likely due to the higher incidence of cirrhosis, non-alcoholic fatty liver disease, and other common causes of liver disease [20,21], our data revealed this correlation but was inconsistent across the three levels of liver dysfunction. Specifically, only the mild fibrosis (0.5 < APRI ≤ 0.7) and significant fibrosis (0.7 < APRI ≤ 1) cohorts were found to have a statistically significant correlation with the male gender. No significant correlation was found between cirrhosis (APRI > 1) and male gender in comparison to the reference group, possibly due to the small sample size (n = 173) of the cirrhosis group. 

Identifying the presence and severity of liver disease preoperatively is imperative because it is a well-established predictor of worse outcomes following orthopedic surgeries. For instance, in patients undergoing TJA, preoperative chronic liver disease is associated with an increased risk for 30-day and one-year mortality, infections, revision surgeries, readmission, UTIs, dislocation, transfusions, and perioperative complications [11,22-24]. With diminished liver function, neutrophil and monocyte function are impaired, increasing the risk for infection and a range of postoperative complications [25]. Additionally, liver dysfunction affects the synthesis of platelets and clotting factors, leading to impaired coagulation and an increased risk for bleeding complications [25].

Our univariate analysis found an association between cirrhosis (APRI > 1) and major complications and sepsis, while significant fibrosis (0.7 < APRI ≤ 1) was associated with increased rates of myocardial infarction. However, after controlling for other patient demographic and comorbidity factors, neither liver fibrosis nor cirrhosis as indicated by APRI values independently predicted 30-day postoperative outcomes after aRCR. Other studies have shown APRI, and thus liver disease, to be a predictor for poorer outcomes following other orthopedic procedures [12].

The lack of independent association between liver dysfunction and postoperative complications in this study may be due to the nature of the aRCR procedure, which is less invasive compared to non-arthroscopic orthopedic surgeries such as TJA or tibia fracture fixation. Several studies have found a lower risk of complications following aRCR compared to open orthopedic surgeries [26,27], which may indicate that aRCR inherently yields a weaker association with early postoperative complications.

The findings of this study should be interpreted in the context of its limitations. Firstly, the study used data obtained from the ACS-NSQIP database, which may have inherent limitations related to its variables as well as limiting our ability to study non-complication variables of interest such as postoperative function. Secondly, the retrospective nature of the study introduces its own potential biases. It limits the ability to establish temporal relationships between liver fibrosis or cirrhosis and 30-day postoperative outcomes. A prospective study with a standardized protocol and longitudinal follow-up greater than the present study’s 30-day follow-up period may provide more robust evidence in the future. Next, because current guidelines recommend that patients should only undergo preoperative liver function testing prior to non-hepatic surgery if they have a history, clinical suspicion, or significant risk factors of liver disease [13], the study population consists of both patients with and without previously diagnosed liver disease. Since the database contains limited information related to patients’ past medical history, we were unable to separate patients with and without previous liver disease-related diagnoses to investigate APRI as a screening tool versus as a confirmatory test. A significant portion of the aRCR population was excluded for missing preoperative liver function tests. This may be partly due to the low invasiveness of the aRCR procedure which does not require as comprehensive of preoperative laboratory testing compared to more invasive procedures, and partly due to excluded patients having a low suspicion of liver dysfunction. Therefore, there may exist some degree of selection bias in our study population. Finally, while APRI is a validated index for liver fibrosis assessment, it has its own limitations, including variability among different etiologies of liver disease and its classification as a surrogate marker to assess the presence of liver disease [16,28-30]. 

## Conclusions

Among those with predicted liver damage based on preoperative APRI, 30-day postoperative complications following aRCR were not found to be independently associated with preoperative mild fibrosis, significant fibrosis, or cirrhosis. These results suggest that in the preoperative risk stratification of aRCR, abnormal preoperative APRI should not be considered a strong deterrent in surgical candidate selection.
